# Trends in the Levels of Serum Lipids and Lipoproteins and the Prevalence of Dyslipidemia in Adults with Newly Diagnosed Type 2 Diabetes in the Southwest Chinese Han Population during 2003–2012

**DOI:** 10.1155/2015/818075

**Published:** 2015-05-18

**Authors:** Jing Tian, Hewen Chen, Fang Jia, Gangyi Yang, Shengbing Li, Ke Li, Lili Zhang, Jinlin Wu, Dongfang Liu

**Affiliations:** Department of Endocrinology and Metabolism, The Second Affiliated Hospital of Chongqing Medical University, 76 Linjiang Road, Yuzhong District, Chongqing 400010, China

## Abstract

*Objective*. To determine the trends of serum lipid levels and dyslipidemia in adults newly diagnosed with type 2 diabetes mellitus during 2003–2012 in Southwest China. *Methods*. Serum lipid measurements of 994 adults were obtained from 5 independent, cross-sectional studies (2003-2004, 2005-2006, 2007-2008, 2009-2010, and 2011-2012). The main outcome measures were mean serum total cholesterol, low-density lipoprotein cholesterol, high-density lipoprotein cholesterol, and triglyceride levels; body mass index; hemoglobin A1C level; and the percentages of patients with dyslipidemia, hypertension, coronary heart disease, and cerebrovascular disease. *Results*. The mean total cholesterol and low-density lipoprotein cholesterol levels increased from 4.92 ± 1.15 to 5.30 ± 1.17 mmol/L (*P* = 0.039) and 2.72 ± 0.83 to 3.11 ± 1.09 mmol/L (*P* = 0.004), respectively, and the mean HDL cholesterol level declined from 1.22 ± 0.30 to 1.06 ± 0.24 mmol/L (*P* < 0.001). The percentages of patients with dyslipidemia increased gradually. The incidence of coronary heart and cerebrovascular diseases increased from 8.2% to 19.1% and 6.6% to 15.3%, respectively (*P* < 0.05). *Conclusion*. Unfavorable upward trends were observed in serum lipid levels and the prevalence of dyslipidemia, coronary heart disease, and cerebrovascular disease in adults newly diagnosed with type 2 diabetes mellitus in Southwest China during 2003–2012.

## 1. Introduction

In recent years, owing to the rapid economic development and changes in lifestyle, the number of patients with diabetes mellitus in China has increased dramatically. The age-standardized prevalence rates of total diabetes and prediabetes in China are reportedly 9.7% and 15.5% [1]. Among the patients with type 2 diabetes mellitus, cardiovascular disease is the most important cause of mortality and morbidity. Epidemiologic studies have demonstrated that dyslipidemia increases the risk of cardiovascular diseases [2–4]. Because dyslipidemia is asymptomatic, it is difficult to diagnose and manage, and many clinicians ignore the importance of dyslipidemia in patients with diabetes.

A previous study based on the Chinese national nutrition and health survey in 2002 investigated the prevalence of dyslipidemia in 14252 Chinese adults and found that the overall prevalence of dyslipidemia was 18.6% (22.2% in men and 15.9% in women) [5]. In addition, although numerous studies have shown that serum lipid levels are high in diabetes patients, data on serum lipid and lipoprotein levels in patients newly diagnosed with type 2 diabetes mellitus are scarce, especially in Chongqing, a midlevel developed city in Southwest China with living conditions representative of the majority of Chinese cities and thus a fitting representative of the general Chinese population.

The aim of the study was to analyze the trends in serum lipid levels and the prevalence of patients with dyslipidemia, coronary heart disease (CHD), hypertension, and cerebrovascular disease in adults with newly diagnosed type 2 diabetes mellitus in the Southwest Chinese Han population during 2003–2012.

## 2. Materials and Methods

### 2.1. Patients and Data Collection

Overall, 994 inpatients newly diagnosed with type 2 diabetes mellitus between January 2003 and December 2012 were collected from 3 different hospitals including The Second Affiliated Hospital of Chongqing Medical University, Bishan District People's Hospital of Chongqing, China, and Fuling District People's Hospital of Chongqing, China. All participants were Han ethnic. None of these patients received prescribed lipid or hormone replacement therapy, and those taking lipid-lowering medication were excluded. All subjects had complete data regarding total cholesterol (TC), low-density lipoprotein cholesterol (LDL-C), high-density lipoprotein cholesterol (HDL-C), and serum triglyceride (TG) levels from each study year. This project was approved by the Human Research Ethics Committee of Chongqing Medical University. Because our study is retrospective, patient information was anonymized and deidentified when data were collected and analyzed, thus exempting the requirement for informed consent.

### 2.2. Diagnostic Criteria

According to National Cholesterol Education Program Adult Treatment Panel (NCEP ATP) III [6], the diagnosis of dyslipidemia for patients with diabetes is based on the presence of one or more of the following criteria: high TC (≥5.18 mmol/L), high LDL cholesterol (≥2.60 mmol/L), low HDL cholesterol (≤1.04 mmol/L for men and ≤1.30 mmol/L for women), and high TG (≥1.70 mmol/L) levels. Hypertension was defined as systolic and diastolic blood pressure ≥140 and ≥90 mmHg, respectively, and/or current treatment with antihypertensive medications. CHD was diagnosed on the basis of a history of acute coronary syndrome or was confirmed by computed tomography angiography, or coronary angiography. Cerebrovascular disease, including ischemic stroke and hemorrhagic stroke, was diagnosed using brain computed tomography or brain magnetic resonance imaging.

### 2.3. Design and Measurements

The trends of the mean serum lipid levels were analyzed by comparing the data of 30–89-year-old adults obtained from 5 studies: 2003-2004, 2005-2006, 2007-2008, 2009-2010, and 2011-2012. These 5 sets of data were obtained independent of each other.

At the time of diagnosis, blood was drawn after an 8 h fast for measuring TG, TC, HDL-C, LDL-C, and hemoglobin A1C (HbA1C) levels. The same methods of lipid level measurements were used in all 5 examinations. The GPO-PAP method and COD-CE-PAP assay were used to determine triglyceride and total cholesterol levels, respectively. HDL and LDL cholesterol levels were measured using the CAT method (Maker Biotechnology Co., Ltd., Sichuan, China). The HbA1C level was measured using a high performance liquid chromatography method. The body mass index (BMI) was calculated by dividing the weight (kg) by the square of the height (m^2^). Information about the diagnosis of CHD, hypertension, and cerebrovascular disease was collected from the patients' medical records.

### 2.4. Statistical Analyses

The results are expressed as mean ± standard deviation for TC, HDL-C, and LDL-C. Geometric means and standard errors of geometric means are presented for serum triglyceride levels because the distribution is highly skewed. The prevalence of dyslipidemia and the morbidity rates of CHD, hypertension, and cerebrovascular disease are presented as percentages. We calculated the differences between the time periods using analysis of variance (ANOVA) for continuous variables; Kruskal-Wallis test was used for nonnormally distributed variables; to test whether the percentage of patients with dyslipidemia, hypertension, CHD, and cerebrovascular disease changed over the years, the chi-square test was used. All data were analyzed using SPSS version 18.0 (SPSS Inc., Chicago, IL, USA), and *P* values less than 0.05 were considered statistically significant.

## 3. Results

The patient characteristics are shown in Table 1. The medical data of 994 subjects (550 men and 444 women; age range: 30–89 years) were analyzed. The time trends in the mean age (*P* = 0.723), BMI level (*P* = 0.052), and haemoglobin A1c (HbA1C) level (*P* = 0.391) of the patients with newly diagnosed type 2 diabetes were not of statistical significance during the study period (Table 1).

### 3.1. Trends in Serum Total Cholesterol and LDL Cholesterol Levels

**Figure 1:**
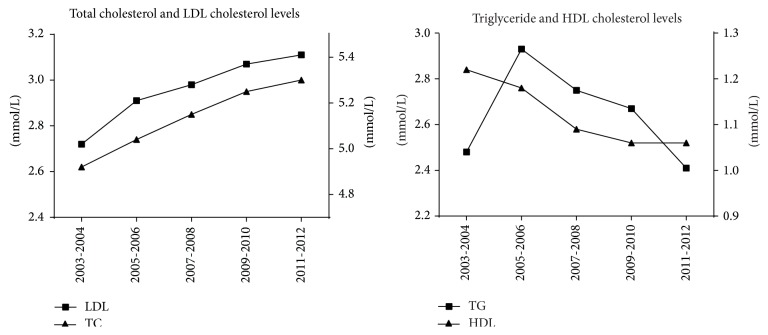
Trends in serum total cholesterol, LDL cholesterol, triglyceride, and HDL cholesterol levels between 2003 and 2012.

The mean TC level of 30–89-year-old adults increased from 4.92 ± 1.15 mmol/L in 2003-2004 to 5.30 ± 1.17 mmol/L in 2011-2012 (*P* = 0.039; Figure 1). No significant differences were observed when men and women were analyzed separately. The mean serum TC level was higher in women than in men in each period (Table 2). The mean LDL-C levels gradually increased between 2003-2004 and 2011-2012 (*P* = 0.004; Figure 1). A significant increase was observed in the LDL-C levels in men (from 2.64 ± 0.80 mmol/L in 2003-2004 to 3.09 ± 1.08 mmol/L in 2011-2012; *P* = 0.001; Table 2). In women, the LDL-C level increased from 2.81 ± 0.86 mmol/L in 2003-2004 to 3.18 ± 1.26 mmol/L in 2011-2012, but this increase was not statistically significant (*P* = 0.280; Table 2). In each period, the mean LDL-C level was higher in women than in men (Table 2).

### 3.2. Trends in Triglycerides and HDL Cholesterol Levels

The mean serum triglyceride levels of all patients increased till 2005-2006 and then decreased till 2011-2012 (*P* = 0.148; Figure 1). The mean HDL-C level decreased continuously from 1.22 ± 0.30 mmol/L in 2003-2004 to 1.06 ± 0.24 mmol/L in 2011-2012 (*P* < 0.001; Figure 1). In men, the HDL cholesterol level showed a significant (*P* < 0.001) downward linear trend from 1.19 ± 0.36 mmol/L in 2003-2004 to 1.02 ± 0.22 mmol/L in 2011-2012 (Table 2), whereas, in women, the HDL-C levels showed no significant change. The mean serum HDL-C level was lower in men than in women in each period (Table 2).

### 3.3. Prevalence of Dyslipidemia, Hypertension, CHD, and Cerebrovascular Disease

**Figure 2:**
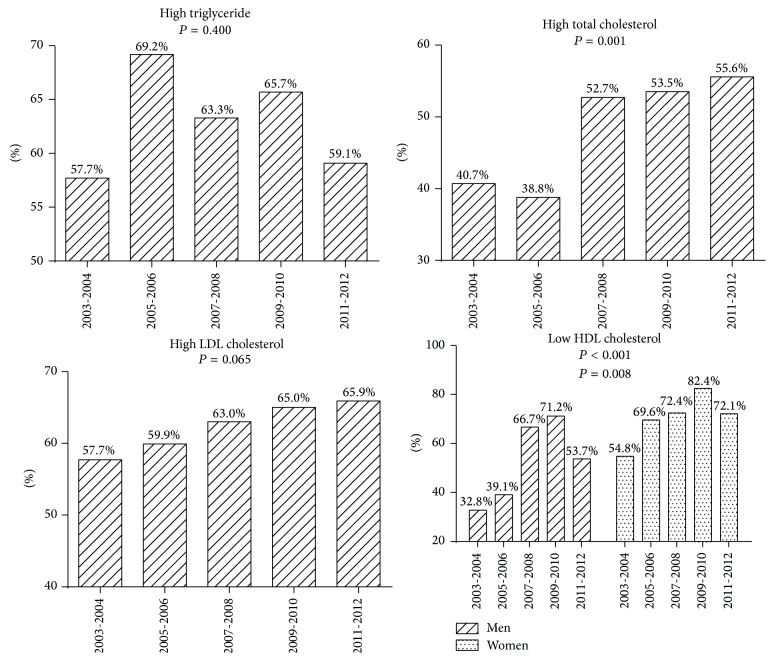
The proportions of patients with dyslipidemia during 2003–2012.

The percentage of patients with a serum total cholesterol level of at least 5.18 mmol/L increased gradually from 40.7% in 2003-2004 to 55.6% in 2011-2012 (*P* = 0.001; Figure 2). The prevalence of hypo-HDL-cholesterolemia for men increased greatly from 32.8% in 2003-2004 to 71.2% in 2009-2010 and then decreased to 53.7% in 2011-2012 (*P* < 0.001; Figure 2). In women the percentage of hypo-HDL-cholesterolemia increased from 54.8% to 72.1% during the study period (*P* = 0.008; Figure 2). No significant differences were observed in the prevalence of hyper-LDL-cholesterolemia and hypertriglyceridemia between 2003 and 2012. But we observed that majority of patients newly diagnosed with type 2 diabetes mellitus have high LDL cholesterol levels.

**Figure 3:**
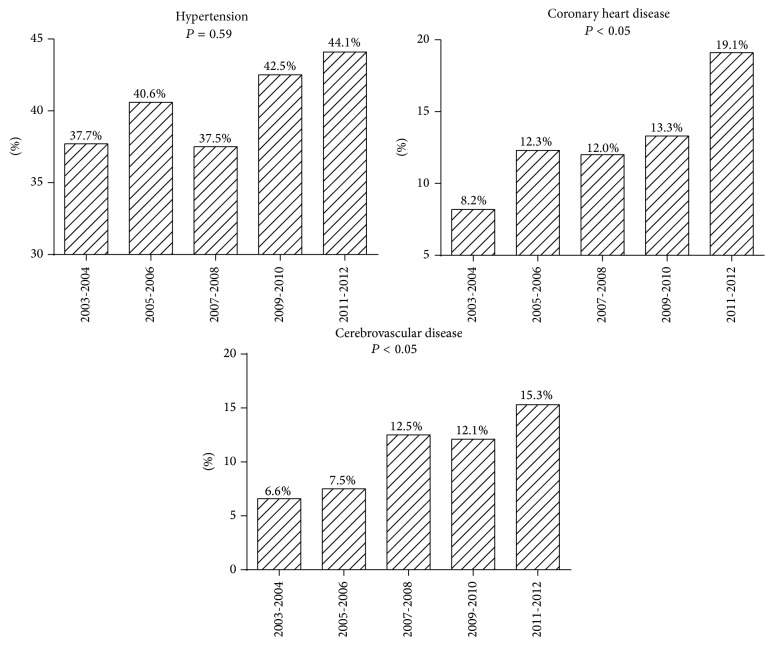
Percentages of patients with hypertension, coronary heart disease, and cerebrovascular disease between 2003 and 2012.

The prevalence rates of hypertension, CHD, and cerebrovascular disease at the time of diagnosis of type 2 diabetes mellitus are shown in Figure 3.

## 4. Discussion

In this study, we found that both the TC levels and the percentage of patients with hypercholesterolemia increased gradually, and the same trend was observed for LDL cholesterol. The NCEP ATP III constantly monitors the elevated levels of LDL cholesterol, which is a major risk factor contributing to CHD [7]. The recommended LDL cholesterol levels for individuals at high risk for cardiovascular disease and for those without overt cardiovascular disease are <1.8 mmol/L and <2.6 mmol/L, respectively [8]. In our study, the lowest mean LDL cholesterol level for all participants was 2.72 ± 0.83 mmol/L observed in 2003-2004 and not only was higher than the recommended levels, but was also found to have increased since 2003-2004, reaching a high of 3.11 ± 1.09 mmol/L in 2011-2012. Hence, the management of high LDL cholesterol level in diabetes patients is vital for reducing the risk of cardiovascular events.

Although lowering LDL cholesterol level is the major focus in this field of study, benefits associated with targeting other lipids have also been previously demonstrated. For example, HDL cholesterol level is a strong negative indicator for cardiovascular events, with high levels of HDL cholesterol having been suggested to protect against atherosclerotic manifestations. On the basis of several epidemiological studies, when HDL cholesterol levels are increased by 1.0 mg/dL, the occurrence of CHD simultaneously decreases by 2-3% [9]. In our study, unfavorable upward trends were observed in HDL cholesterol levels and the prevalence of low HDL cholesterol for men and women. Accordingly, several studies have shown that HDL cholesterol levels were markedly reduced in both men and women with diabetes mellitus compared with those in nondiabetic individuals [10]. Therefore, management of HDL cholesterol levels should be essential in patients with newly diagnosed type 2 diabetes mellitus.

Although no significant changes were observed in the serum triglycerides levels during 2003–2012, we speculate that this trend may have been influenced by differences in the diets of the patients. According to the values of NCEP ATP III classification, both men and women had elevated triglyceride levels in each time period. Being overweight, sedentary, or a cigarette smoker is associated with elevated triglyceride levels; thus, positive lifestyle changes can help lower triglyceride levels [11].

In our study, we found that, at the time of diagnosis of diabetes, elevated triglyceride levels, high LDL cholesterol levels, and low HDL cholesterol levels were commonly noted, and these are all characteristic features of diabetic dyslipidemia [12]. Moreover, we found that the mean total and LDL cholesterol levels were higher in women than in men. One possible reason for this observation is that hormonal changes after menopause cause a rapid increase in lipid levels, which become higher than those in men [13].

In the past several years, total serum cholesterol levels have declined in most Western populations, such as in Australia, North America, and Europe; however, they have increased in the East, Southeast Asia, and the Pacific regions [14, 15]. In China, the mean levels of serum lipids have been reported to be much higher in areas with rapid economic growth, such as Shanghai and Beijing, compared to those in rural areas [16]. Remarkable socioeconomic development along with a rise in the living standards of Chinese Han population in the past few decades may explain the unfavorable trends in the lipid profiles observed herein. Numerous studies have indicated that lifestyle factors such as high calorie intake, population-wide sedentary lifestyle, decreased physical activities, and increased obesity are related to increased serum lipids levels [17, 18]. Although we did not investigate changes in the eating habits and physical activities of individuals in this study, other studies have reported that the eating habits of the Chinese population have changed dramatically from the traditional dietary pattern (plant-based, low-fat diet) to the Western dietary pattern (high-fat, low-carbohydrate diet), which is characterized by excess calorie intake [19]. Another study reported that China is experiencing rapidly escalating rates of overweightness and obesity in recent years [20]. In China, the rate of overweightness has doubled from 13.5% in 1991 to 26.7% in 2006, and the number of obese individuals has tripled [21]. The classifications of overweightness and obesity in Chinese individuals are based on the BMI cutoffs of 24 kg/m^2^ and 28 kg/m^2^, respectively [22], and in our study, the average BMI was higher than 24 kg/m^2^ in each period, indicating that most of the patients with newly diagnosed type 2 diabetes mellitus were overweight.

Another possible factor responsible for the changes in serum lipid levels is a decrease in physical activity. A sedentary lifestyle is now becoming widespread, especially among urban residents, with the average weekly physical activity among adults having decreased by more than a third between 1991 and 2006 [23]. Increasing reliance on automobiles and increasing availability of buses have replaced the traditional ways of transportation such as biking or walking, and the increased use of television and computers has replaced the traditional means of recreation such as swimming and running [24, 25]. Thus, lifestyle and behavioral changes need to be addressed in order to reduce the incidence of dyslipidemia.

The United Kingdom Prospective Diabetes Study provided conclusive evidence that, with effective control of hyperglycemia in diabetes patients, morbidity due to microvascular complications can be significantly reduced. However, intensive treatment of hyperglycemia did not significantly reduce macrovascular disease [26]. In our study, in accordance with the increase in serum lipid levels, the trends of CHD and cerebrovascular disease in people newly diagnosed with diabetes also continuously increased during the study period. Hence, immediate measures should be taken to prevent macrovascular disease in this population.

The results of this study should be interpreted in light of the following limitations. First, when we classified the study participants into different ages, no statistical significance was observed, and this could be because we did not use a very large enough sample size. Second, this was a cross-sectional analysis and we could investigate factors affecting the prevalence of dyslipidemia, such as dietary intake and physical activities. Hence, further research is needed to assess the effects of lifestyle factors on serum levels of lipids. Third, the patients taking lipid-lowering medication were excluded; the percentages of people with dyslipidemia did not include these patients.

## 5. Conclusion

The results of this study indicated unfavorable upward trends in serum lipid levels and in the prevalence of dyslipidemia, CHD, and cerebrovascular disease among patients with newly diagnosed type 2 diabetes mellitus in the Southwest Chinese Han population. Widespread promotions of ways to lower lipid levels and of treatment strategies should be intensified to reduce cardiovascular morbidity and mortality. In conclusion, dyslipidemia is preventable and now is the ideal time for implementing appropriate strategies to battle this epidemic.

## Figures and Tables

**Table 1:** Patient characteristics.

	2003-2004	2005-2006	2007-2008	2009-2010	2011-2012
Sample sizes	122	212	184	240	236
Men	61	128	108	132	121
Women	61	84	76	108	115
Mean age (years)	56.89 ± 12.87	57.35 ± 13.00	56.24 ± 12.42	56.00 ± 12.13	55.90 ± 11.93
BMI (kg/m^2^)	25.12 ± 2.14	25.18 ± 2.64	25.19 ± 2.96	25.20 ± 2.08	24.58 ± 2.94
HbA1C (%)	10.25 ± 1.22	10.16 ± 2.37	10.64 ± 2.32	10.26 ± 3.10	10.27 ± 2.60

Values are expressed as means ± SD. BMI: body mass index; HbA1c: haemoglobin A1c.

**Table 2:** Serum lipids and lipoproteins levels in patients with newly diagnosed type 2 diabetes in Chongqing, 2003–2012.

		03~04	05~06	07~08	09~10	11~12	*P*
TC (mmol/L)	Total	4.92 ± 1.15	5.04 ± 1.37	5.15 ± 1.46	5.25 ± 1.19	5.30 ± 1.17	0.039
	Men	4.81 ± 1.20	4.90 ± 1.29	5.13 ± 1.51	5.12 ± 1.15	5.26 ± 1.19	0.056
	Women	5.03 ± 1.09	5.40 ± 1.83	5.19 ± 1.38	5.50 ± 1.28	5.37 ± 1.40	0.247

TG (mmol/L)	Total	2.48 ± 2.02	2.93 ± 2.63	2.75 ± 2.50	2.67 ± 1.91	2.41 ± 2.08	0.148
	Men	2.72 ± 2.32	3.10 ± 2.82	3.19 ± 2.96	2.95 ± 2.30	2.76 ± 2.45	0.659
	Women	2.24 ± 1.67	2.64 ± 2.28	2.14 ± 1.48	2.34 ± 1.22	2.03 ± 1.50	0.127

HDL (mmol/L)	Total	1.22 ± 0.30	1.18 ± 0.45	1.09 ± 0.45	1.06 ± 0.29	1.06 ± 0.24	<0.001
	Men	1.19 ± 0.36	1.14 ± 0.32	1.00 ± 0.35	1.01 ± 0.29	1.02 ± 0.22	<0.001
	Women	1.26 ± 0.24	1.24 ± 0.59	1.18 ± 0.54	1.11 ± 0.28	1.12 ± 0.26	0.091

LDL (mmol/L)	Total	2.72 ± 0.83	2.91 ± 1.04	2.98 ± 1.02	3.07 ± 0.74	3.11 ± 1.09	0.004
	Men	2.64 ± 0.80	2.77 ± 1.00	2.95 ± 0.98	2.96 ± 0.78	3.09 ± 1.08	0.001
	Women	2.81 ± 0.86	3.13 ± 1.08	3.03 ± 1.08	3.12 ± 0.84	3.18 ± 1.26	0.280

Values are expressed as means ± SD. TC: total cholesterol; TG: triglyceride; HDL: high- density lipoprotein; LDL: low-density lipoprotein.
